# Predicting pulmonary tuberculosis incidence in China using Baidu search index: an ARIMAX model approach

**DOI:** 10.1265/ehpm.23-00141

**Published:** 2023-11-03

**Authors:** Jing Yang, Jie Zhou, Tingyan Luo, Yulan Xie, Yiru Wei, Huanzhuo Mai, Yuecong Yang, Ping Cui, Li Ye, Hao Liang, Jiegang Huang

**Affiliations:** 1 Guangxi Key Laboratory of AIDS Prevention and Treatment, Nanning, China; 2 Guangxi Colleges and Universities Key Laboratory of Prevention and Control of Highly Prevalent Diseases, Guangxi Medical University, Nanning, China; 3 School of Public Health, Guangxi Medical University, Nanning, China; 4 Life Science Institute, Guangxi Medical University, Nanning, China

**Keywords:** PTB, Predictive model, Internet search index, ARIMA model

## Abstract

**Background:**

Existing researches have established a correlation between internet search data and the epidemics of numerous infectious diseases. This study aims to develop a prediction model to explore the relationship between the Pulmonary Tuberculosis (PTB) epidemic trend in China and the Baidu search index.

**Methods:**

Collect the number of new cases of PTB in China from January 2011 to August 2022. Use Spearman rank correlation and interaction analysis to identify Baidu keywords related to PTB and construct a PTB comprehensive search index. Evaluate the predictive performance of autoregressive integrated moving average (ARIMA) and ARIMA with explanatory variable (ARIMAX) models for the number of PTB cases.

**Results:**

Incidence of PTB had shown a fluctuating downward trend. The Spearman rank correlation coefficient between the PTB comprehensive search index and its incidence was 0.834 (*P* < 0.001). The ARIMA model had an AIC value of 2804.41, and the MAPE value was 13.19%. The ARIMAX model incorporating the Baidu index demonstrated an AIC value of 2761.58 and a MAPE value of 5.33%.

**Conclusions:**

The ARIMAX model is superior to ARIMA in terms of fitting and predicting accuracy. Additionally, the use of Baidu Index has proven to be effective in predicting cases of PTB.

**Supplementary information:**

The online version contains supplementary material available at https://doi.org/10.1265/ehpm.23-00141.

## 1. Introduction

PTB is a chronic infectious disease caused by Mycobacterium PTB, which spreads through the population via the respiratory tract [1]. As the largest developing country in the world, China possesses a high burden of Pulmonary Tuberculosis (PTB). According to the 2022 global report on PTB, in 2021, the estimated number of new PTB cases in China was 780,000, with an incidence rate of 55 per 100,000 population. China ranked third among countries with a high burden of PTB in terms of incidence rate [2–4]. Given the country’s large population and high number of PTB infections, China needs to prioritize efforts to reduce the incidence of the disease. This includes intensifying the detection, diagnosis, and treatment of PTB patients, optimizing the PTB prevention and treatment system, and increasing financial investment [5]. Currently, the conventional disease surveillance system is inadequate for accurately predicting and detecting the new cases of PTB. Consequently, it is essential to investigate new monitoring models for PTB.

Since the advent of the big data era, an increasing number of studies have utilized search data for epidemiological research and early warning of infectious diseases. As early as 2009, researchers discovered the effectiveness of combining search data with disease surveillance and early warning through the development of the Google Flu Trends section [6, 7]. Other diseases, such as H7N9, dengue, gonorrhoea, brucellosis, AIDS, and COVID-19, have also been modeled using search engine data [8–10]. However, there remains a lack of studies focusing on the prediction model of PTB using Internet search data, which can be widely implemented for disease surveillance and early warning purposes in China. For long-term monitoring of infectious disease cases, the ARIMA model is commonly used. It offers a relatively simple modeling method, a wide application range, and produces intuitive and easily understood results. It has distinct advantages in terms of popularization, making it easier for government and health department personnel to learn and master. Therefore, the ARIMA model is a suitable supplementary tool for disease surveillance. The ARIMAX model, which combines network information, is an extension of the ARIMA model. By incorporating China’s internet search index, which is more relevant to the country’s actual situation, ARIMAX further enhances the predictability and warning capabilities for PTB incidence in China. This is of great significance for disease monitoring.

As of the end of 2017, the number of internet users in China reached 772 million and continues to grow. Baidu, the most widely used search engine in China, has approximately 86.7% of Chinese users on both mobile and PC platforms [11, 12]. Therefore, the data collected from Baidu Index can accurately reflect the level of concern and understanding of disease spread among Chinese people.

By using Baidu Index and the monthly PTB incidence index, this study established a predictive model that offered a new way for the government and medical institutions to monitor the incidence of PTB. Moreover, this approach addressed some of the shortcomings of traditional monitoring methods.

## 2. Materials and methods

### 2.1 Data sources

**Fig. 1:**
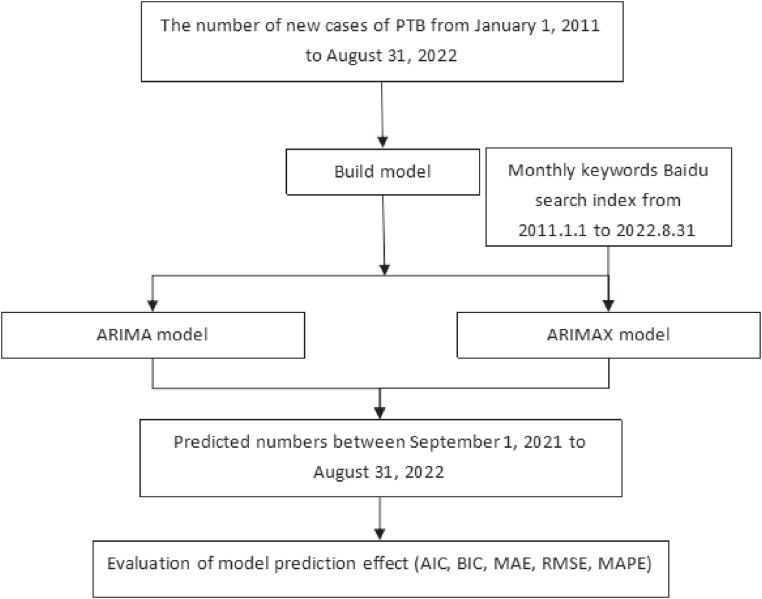
Analysis procedure diagram.

The epidemiological surveillance data used in this study came from the National Health Commission of the People’s Republic of China [13], which published the number of new cases of PTB each month from January 2011 to August 2022 (a total of 140 months). The dataset involved only case statistics, whereas no patients’ personal information or related privacy information was obtained. Additionally, the collected data were devoid of any missing values (Fig. 1).

The search engine data was obtained from Baidu Index [14]. The search index data was collected from January 1, 2011 to August 31, 2022 (Fig. 1). The details were presented in the additional file (Additional file 1).

### 2.2 Keywords selection and filtering

#### 2.2.1 Establish a keyword database

The process of keywords collection consisted of three steps: 1) acquisition and screening. PTB-related group words were obtained by searching “pulmonary tuberculosis (PTB)” in the webmaster’s database where keywords could be crawled [15]; 2) to obtain PTB-related keywords, we searched the “pulmonary tuberculosis (PTB)” keyword demand map on the Baidu Index platform; 3) we combined this information with the latest “Guideline for primary care of pulmonary tuberculosis (2018)” [16] to supplement keywords that may be related to PTB but were not included. After obtaining the preliminary PTB keyword group words, we collected the daily search volume data for each keyword on the Baidu Index platform from January 1, 2011 to August 31, 2020. We then assessed whether the search volume was too low during this period. Any keywords with low search volume or low correlation with PTB were excluded, and a keyword database was established.

Finally, dividing the keywords into five categories: comprehensive category, etiology category, examination category, symptom category, and prevention and treatment category [17–19].

#### 2.2.2 Filtering keywords

This research applied Microsoft Excel 2019 to develop a statistical analysis database. Further descriptive and correlation analyses were then undertaken by SPSS 26.0 software, using a *P* < 0.05 result indicated a statistically significant difference.

Initially, the daily search volume data from Baidu Index were computed to determine the monthly average value. Then, a Spearman rank correlation was performed between the monthly average value of keyword search volume and the monthly new cases of PTB. Keywords with an absolute value of rank correlation coefficient greater than 0.5 and a test value of *P* < 0.05 were considered high correlation keywords [20]. The rest were eliminated.

Secondly, we conducted a time-series cross-correlation analysis. Then, by analysing highest correlation coefficient and its corresponding lag value for each keyword to use in calculating the overall comprehensive index. Keywords with a maximum cross-correlation coefficient of less than 0.5 were excluded. Finally, the keywords used to calculate the PTB comprehensive search index and construct the model were obtained.

#### 2.2.3 The composition of the PTB comprehensive search index

The PTB comprehensive search index was obtained by grouping keywords according to the lag period and calculating the weight and sum, which were then combined with the Spearman correlation coefficient [20, 21]. Its composition method and calculation formula were as follows.

$${\text{Weight}}_{\text{ki}}=\rho_{\text{ki}}/\sum_{\text{i}=1}^{\text{n}}\rho_{\text{ki}}$$ (1)

$$\begin{array}{rl} & {\text{PTB Comprehensive Search Index}}_{\text{k}}\\ & \;=\sum_{\text{i}=1}^{\text{n}}{\text{weight}}_{\text{ki}}{\text{keyword}}_{\text{ki}} \end{array}$$ (2)

In the above two formulas, “k” represents the potential time lag, “n” represents the number of keywords present in each time lag, “ρki” is the Spearman rank correlation coefficient that includes the specific keyword (i) with a particular time lag (k), and “weight_ki_” and “keyword_ki_” represent the weight of the keyword in a specific lag period and its Baidu search index, respectively. The best outcome is achieved when Spearman’s rank correlation index between the PTB comprehensive search index and the number of epidemic cases is greater than 0.8.

### 2.3 Model construction and evaluation

#### 2.3.1 Stabilization of the original time series

To generate time series and analyze data trends, we used the Augmented Dickey-Fuller test (ADF test) and the Ljung-Box Q test to confirm the statistical significance and stationarity of the sequence. In cases where the sequence was non-stationary, techniques such as differentiation, seasonal differentiation could be employed to induce stationarity.

#### 2.3.2 Model construction and estimation

The ARIMA and ARIMAX models were developed using R 4.2.2 software and the forecast package. In the field of public health, the ARIMA model was commonly used to detect outbreaks of infectious diseases and predict the trends of epidemics [22]. The ARIMA (p, d, q) model was an extension of the autoregressive (AR) model, the moving average (MA) model, and the ARMA model [10]. Essentially, the ARIMA model attempted to uncover hidden time series patterns within the data through autocorrelation and data differentiation, and then employed these patterns to make future data predictions. The ARIMAX model, built upon the ARIMA model, incorporated the Baidu index as an external influencing factor and incorporated the time series of disease case numbers for joint modeling [23, 24].

The data was divided into a training dataset covering the period from January 1, 2011, to August 31, 2021 (a total of 128 months), and a test dataset encompassing the period from September 1, 2021, to August 31, 2022 (a total of 12 months), respectively. The common way to divide the training set and test set was to use 80% of the data as the training set and the remaining 20% as the test set. However, because of the large number of actual data, and the prediction time within one year was more in line with the current needs of infectious disease surveillance, its accuracy was also relatively high. Therefore, we used nearly 90% of the data as the training set and nearly 10% of the data as the test set in the actual data. In the cross validation part, we used the partition ratio of the training set with the partition ratio of 0.75/0.8/0.85/0.9. The results indicated that the model’s evaluation index values were superior when the partition ratio was 0.9, and these results remained consistent over 10 repetitions.

Based on the Akaike Information Criterion (AIC), this research used the auto.arima function and the training dataset of PTB case report numbers to obtain the optimal parameters, which will help to construct the optimal ARIMAX model. The optimal parameters for the ARIMAX model were determined using the AIC minimum principle with the training datasets for PTB case report numbers and the composite index. The “least squares method” was used for significance testing of the models [25].

#### 2.3.3 Test of residuals

To confirm whether the residuals of the model conform to a normal distribution, a QQ plot, residual plot, residual auto-correlation function (ACF) plot, and residual partial auto-correlation function (PACF) plot should be generated. Subsequently, the Ljung-Box Q test should be performed to confirm whether the model residuals constitute a white noise sequence. Upon passing the test, predictions can be made.

### 2.4 Model prediction and prediction effect evaluation

We applied ARIMA and ARIMAX models to forecast the number of reported cases of PTB in the provided test dataset. In order to evaluate the accuracy of the model’s predictions, metrics such as mean absolute error (MAE), mean absolute percentage error (MAPE) will be employed.

## 3. Results

### 3.1 Analysis results of the number of PTB cases and keyword Baidu index search data

**Fig. 2:**
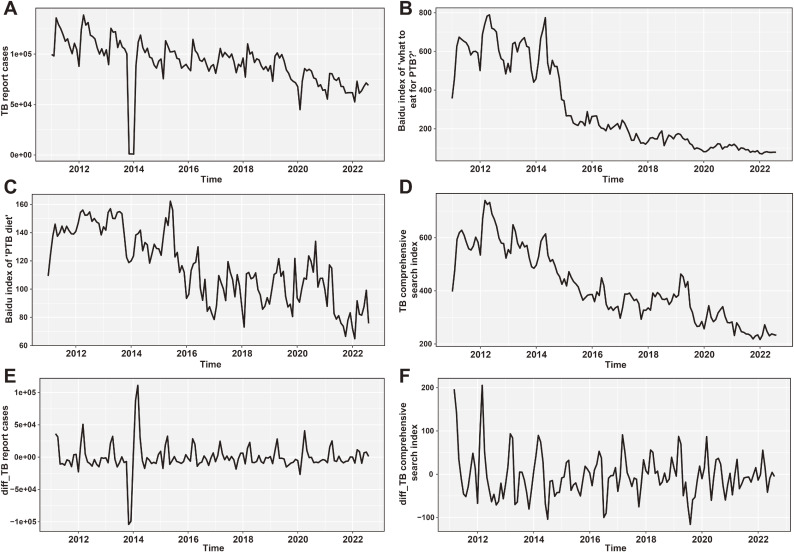
Time Series Plot of PTB Cases and Baidu Search Index of Selected Keywords. A is the time series graph of the reported number of PTB cases in PTB reports. B and C are the time series graphs of the keywords “what to eat for PTB” and “PTB diet” respectively. D is the time series graph of the PTB comprehensive search index. E and F are the time series graphs of the smoothed reported number of PTB cases and the PTB comprehensive search index, respectively.

From January 2011 to August 2022, a total of 25.3858 million cases of PTB were reported in China, with an average annual number of new cases of 1.0041 million. The time series graph depicting the search volume of relevant keywords and the number of PTB cases indicates a positive correlation between the two factors, demonstrating a decline over time (Fig. 2A–C).

A total of 56 keywords have been included in keyword database. These have been divided into five categories, which include 13 comprehensive keywords, 5 etiology keywords, 2 examination keywords, 21 symptom keywords, and 15 prevention and treatment keywords.

#### Correlation analysis between keyword Baidu index and the number of disease cases

We used the Spearman rank correlation method to analyze the correlation between the Baidu Index of keywords and the number of new cases of the disease per month. 23 keywords were selected that exhibit a strong correlation, with “肺结核吃什么好(what to eat for PTB)” having the strongest correlation coefficient of ρ = 0.79 (Table 1).

**Table 1:** Partial monthly Baidu search index for PTB-related keywords

Keywords*	**Mean ± SD**	**Coefficients**	***P* Value**
肺结核 (Pulmonary tuberculosis)	6306.32 ± 1918.36	−0.336	<0.001
淋巴结核 (Lymphatic tuberculosis)	721.22 ± 149.52	0.663	<0.001
肺痨 (Consumption of lung)	456.57 ± 346.55	−0.72	<0.001
肺癌的早期症状和前兆 (Early symptoms and precursors of lung cancer)	2048.98 ± 4239.39	−0.638	<0.001
结核病症状 (Tuberculosis symptom)	284.32 ± 131.06	0.743	<0.001
肺结核会传染吗 (Can PTB be contagious)	1803.27 ± 1211.44	−0.285	0.001
肺结核的潜伏期 (The incubation period of PTB)	167.86 ± 132.09	0.719	<0.001
肺结核饮食 (PTB diet)	115.47 ± 24.85	0.748	<0.001
肺结核吃什么好 (what to eat for PTB)	305.20 ± 224.84	0.79	<0.001

Note: *Categories and keywords are presented in Chinese (English) format.

### 3.2 Cross-correlation analysis and PTB comprehensive search index synthesis

To perform a cross correlation analysis, 23 keywords that had a strong correlation with new cases of PTB were chosen. After excluding keywords with a maximum cross-correlation coefficient less than 0.5, six keywords remained. (Table 2).

**Table 2:** Correlation between the keywords and the number of new cases of PTB

**Keywords**	**Maximum** **correlation** **coefficient**	**Lag**	**Standard** **error**
淋巴结核 (Lymphatic tuberculosis)	0.566	0	0.085
结核病症状 (Tuberculosis symptom)	0.605	0	0.085
肺结核咳血 (PTB hemoptysis)	0.514	−2	0.085
肺结核的潜伏期 (The incubation period of PTB)	0.54	0	0.085
肺结核饮食 (PTB diet)	0.608	0	0.085
肺结核吃什么好 (what to eat for PTB)	0.559	0	0.085

Note: *Keywords are presented in Chinese (English) format.

After applying a weight calculation formula, the PTB comprehensive search index was determined. The analysis revealed a Spearman rank correlation coefficient of 0.834 (*P* < 0.001) between the PTB comprehensive search index and the monthly reported cases of PTB.

### 3.3 Establishment of ARIMA and ARIMAX models

The number of reported cases of PTB was consistently decreasing (Fig. 2A). There was a similar trend in the search keywords for “what to eat for PTB” (Fig. 2B) and “PTB diet” (Fig. 2C), as well as the composite index (Fig. 2D), but with some fluctuations, indicating non-stationarity. However, the ADF test showed that the time series for reported PTB cases is stationary (*P* < 0.05) while the composite index was non-stationary (*P* = 0.09725). After applying differentiation lagged at two, both time series became stationary with ADF tests showing *P* values below 0.05 (Fig. 2E and F). The Box-Ljung test indicated that reported PTB cases were suitable for modeling and future disease prediction (*P* < 0.05).

After obtaining stationary time series, the data was split into a training dataset from January 2011 to August 2021 for modeling and a testing dataset from August 2021 to August 2022 for predicting and evaluating model performance.

**Fig. 3:**
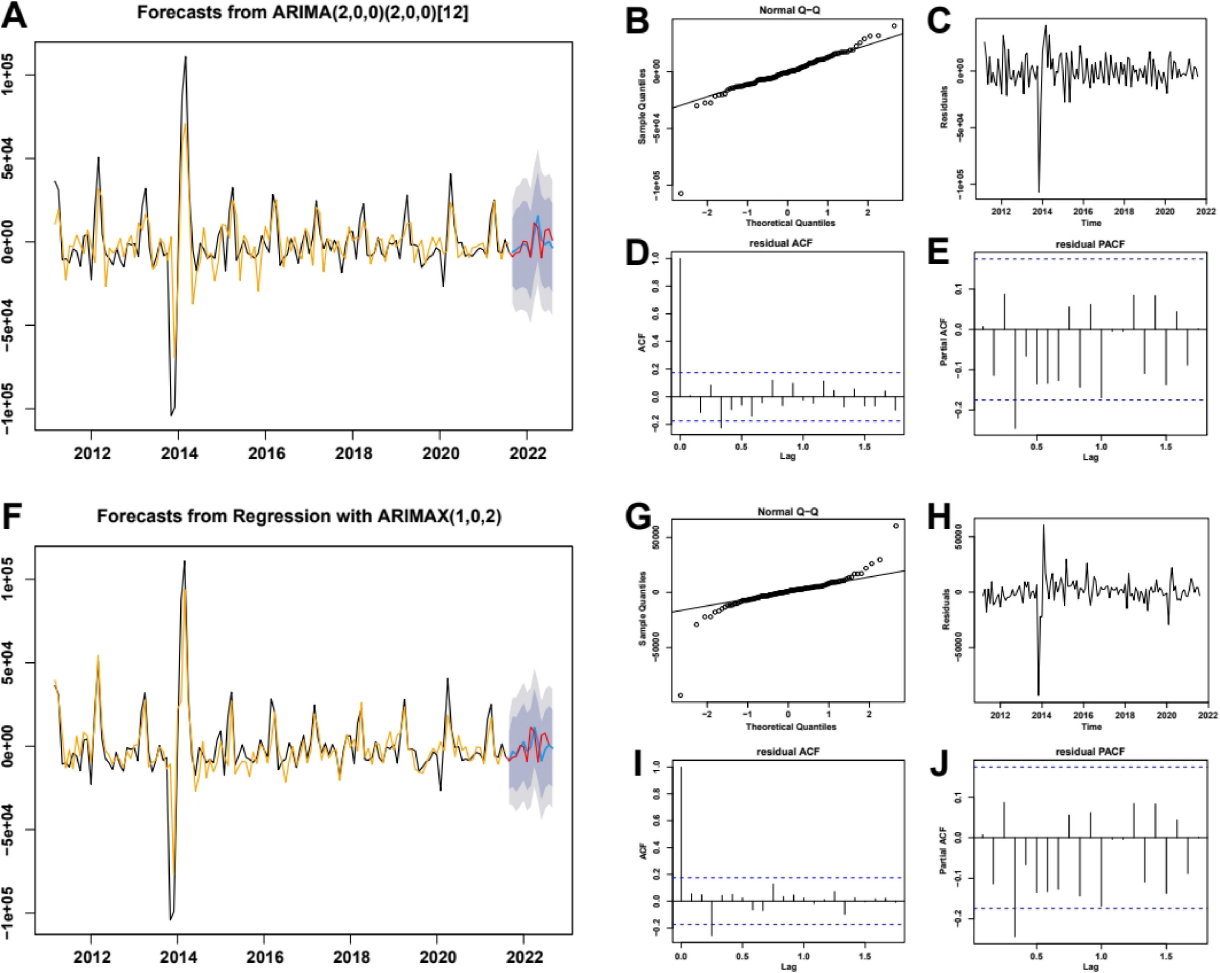
ARIMA and ARIMAX Model Fitted and Forecasted. ARIMA (A–E) and ARIMAX (F–J) model fitting (A and F), residual analysis (B–E, G–J), and prediction performance (A and F) are presented in graphical form. In A and F, the black line represents the true values in the training set. B and G are QQ plots, C and H are residual plots, D and I are ACF plots, and E and J are PACF plots (the blue line represents the confidence interval).

Based on the time series of reported PTB cases and the principle of minimum AIC, the auto.arima function determined to the optimal ARIMA model parameters. The optimal ARIMA model was ARIMA(2,0,0) (2,0,0) [12] (Fig. 3A). The ARIMAX model used a joint modeling approach with time series data of reported PTB cases and the PTB comprehensive search index [24]. The optimal ARIMAX model was determined based on the AIC minimum principle using the auto.arima function and constructed accordingly. The optimal ARIMAX model was ARIMA(1,0,2) (Fig. 3F). Using the “least squares method” for model estimation, both models were significant (*P* < 0.05). Residual analysis show that the residuals of both models followed a normal distribution and the points on the QQ plot were close to the baseline (Fig. 3B, 3G). The residual white noise test (Ljung-Box Q test) returned results of *P*_ARIMA_ = 0.9343 and *P*_ARIMAX_ = 0.5266 respectively, and the residual plot fluctuates near zero (Fig. 3C, 3H). The autocorrelation of the residual (ACF, Fig. 3D, 3I) and partial autocorrelation (PACF, Fig. 3E, 3J) were mostly within the confidence interval (two blue dotted lines). This indicated that the residuals of both the ARIMA model and ARIMAX model were white noise. Both models passed the test and can be used for prediction.

### 3.4 Prediction effectiveness and evaluation of ARIMA and ARIMAX models

After applying the ARIMA and ARIMAX models to forecast the number of reported PTB cases in the test set, it was evident that the predicted values were generally in agreement with the actual values. Furthermore, the actual values were within the range of the predicted interval (Fig. 3A, 3F). To summarize, the output of the model closely aligned with real-world data, implying its reliability and accuracy. By calculating the performance indicators of the two models mentioned above (Table 3), it can be seen that the ARIMAX model was superior to the ARIMA model.

**Table 3:** Model fitting and prediction performance comparison

**Model**	**AIC**	**BIC**	**MAE**	**RMSE**	**MAPE(%)**
ARIMA	2804.41 (2800.49, 2808.33)*	2818.59 (2814.67, 2822.51)*	6286.70 (2356.50, 10216.89)*	7229.86 (3299.66, 11160.05)*	13.19 (−9.62, 35.99)*
ARIMAX	2761.58 (2758.38, 2764.78)*	2775.76 (2772.56, 2778.96)*	4095.75 (1407.84, 6783.65)*	4944.58 (2256.67, 7632.48)*	5.34 (−3.15, 13.83)*

Note: *The numerical value within the parentheses represents the 95% confidence interval.

## 4. Discussion

Official data on infectious disease epidemics in China is predominantly generated through traditional surveillance systems. However, such monitoring systems often suffer from delayed reporting deadlines and inadequate capacity to detect patients comprehensively. In contrast, an Internet search monitoring system may help overcome the report delays and omissions that are common in traditional surveillance systems [26]. Since 2013, researchers have leveraged Baidu Index to analyze epidemic trends and develop models to predict the development of future diseases [21, 27]. Therefore, this study aims to improve PTB monitoring by exploring Baidu Index’s predictive ability for the national PTB epidemic situation.

The study’s established keyword library revealed that the most frequently used keyword was “symptoms of PTB,” which was recorded 21 times. Despite the prevalence of the internet and advances in medical technology, there is still a lack of knowledge regarding PTB among the general population. Thus, it is crucial to promote awareness of PTB symptoms for early detection, diagnosis, and treatment to prevent the spread of the disease.

For long-term surveillance data on the incidence of infectious diseases, the ARIMA model is commonly employed. In some studies comparing prediction models for influenza and COVID-19 surveillance based on network search data, the ARIMA model was found to have lower prediction results compared to the Long Short-term Memory (LSTM) model, Mixed Logistic Regression (MLR) model, and Support Vector Regression (SVR) model in some evaluation indicators. However, it offers simplicity and feasibility in operation and has a wider range of applications. Other models are more susceptible to prediction bias due to missing data and errors in processing [8, 28, 29]. Additionally, the ARIMA model is suitable for various types of time series data as it can effectively capture long-term trends and periodicity, and it can also incorporate various external variables.

The ARIMAX model demonstrated better predictive capability compared to the ARIMA model. Currently, there are several prediction models available, but only a few consider both accuracy and application. Among these models, the ARIMAX model stands out as a simpler and more intuitive option, making it particularly suitable for long-term disease monitoring predictions. Its simplicity makes it easier for a wider audience to learn and apply. Additionally, when combined with the Baidu index, it significantly enhanced prediction accuracy and aligned better with the real-life situation in China. This had significant implications for PTB surveillance and early warning in the country.

Models that incorporated search data, such as Baidu Index, showing improved accuracy compared to those that do not [21, 30]. This trend was observed in infectious diseases such as H7N9 influenza, AIDS, syphilis, and gonorrhea [11, 31–33]. This approach went beyond relying solely on official statistics.

The model obtained in this study exhibited better predictive ability compared to previous studies that also utilized the Baidu search index to establish models for predicting the prevalence of infectious diseases [26, 27, 30, 33]. For example, Ruonan Huang et al. developed a model to predict the incidence rates of HIV/AIDS, syphilis, and gonorrhea by using the Baidu search index. Their results showed mean absolute percentage errors (MAPEs) of 9.39%, 10.11%, and 9.35%, respectively [27]. Similarly, a study on the relationship between the incidence of PTB in Jiangsu Province, China, and the Baidu search index yielded a MAPE value of 8.86% for the fitted neural network model [30]. In contrast, our study utilized the comprehensive search index of PTB to fit the ARIMAX model, resulting in a superior MAPE value of 5.34% compared to the aforementioned studies.

However, there are still some limitations. Firstly, there may be relevant data that we missed due to incomplete keyword inclusion on the Baidu Index. Secondly, the existing data is basic and lacks characteristic information about the population, such as age and profession. Thirdly, the study only focuses on data from China, so the relationship between search queries and HIV diagnoses may differ in other cultural contexts. Another limitation is that the monthly case numbers and search indices analyzed may not fully represent the actual incidence and search volume, as some data were not included. Lastly, relying solely on search engine data may result in incomplete outcomes. Therefore, it is crucial to further explore the correlation between other internet data and PTB epidemic surveillance [34].

In terms of the practical application of the ARIMAX prediction model, there has not been a specific pilot program implemented in China for supplementary monitoring. However, we can discuss its feasibility in theory. In practice, upon obtaining the prediction model results, the implementation of surveillance and prevention mainly revolves around evaluating the predicted number of disease cases. This is followed by targeted disease prevention campaigns, epidemic control measures (such as disinfection, elimination of infection sources, cutting off transmission routes, and protecting susceptible populations), and reinforcing relevant medical resources. These measures allow for prompt response actions prior to a potential epidemic outbreak, and even to prevent the early stages of an outbreak.

While the specific details of an outbreak may vary from one location to another, the fundamentals of prevention prevention measures remain the same. Currently, the mobilization of medical resources in different regions of China primarily relies on the coordination efforts of the national health department. Once the prediction results indicate a potential outbreak in a certain area, China’s epidemic reporting system can promptly and effectively communicate the specific situation, enabling the affected area to swiftly receive the necessary medical resource support.

## 5. Conclusions

In conclusion, this study has revealed that the ARIMAX model, when combined with the Baidu search index, outperforms the traditional ARIMA model in the accurate prediction of the PTB cases’ number. This finding is promising for the field of infectious disease monitoring, as it suggests that Internet search data can serve as a cost-effective and innovative tool for detecting potential PTB patients at an early stage. This study has opened a new avenue for improving disease monitoring, prediction, and prevention in the future.

## Abbreviations

PTB: pulmonary tuberculosis
ARIMA: autoregressive integrated moving average
ARIMAX: ARIMA with explanatory variable
AIC: akaike information criterion
ADF: augmented dickey-Fuller
ACF: auto-correlation function
PACF: partial auto-correlation function
MAE: mean absolute error
MAPE: mean absolute percentage error
RMSE: root mean square error
BIC: bayesian information criterion
LSTM: Long Short-term Memory
MLR: Mixed Logistic Regression
SVR: Support Vector Regression

## Supplementary information

**Additional file 1:** The monthly number of pulmonary tuberculosis cases and 56 key words Baidu index data from January 2011 to August 2022.

## Declarations

### Availability of data and material

The data of PTB cases analyzed in this study were obtained from National Health Commission of the People’s Republic of China (http://www.nhc.gov.cn/). The search engine data was obtained from Baidu Index (https://index.baidu.com/v2/index.html#/).
